# Editorial: Targeting intra- and extracellular signals contributing to cancer stemness and metastasis in aggressive cancers, Volume II

**DOI:** 10.3389/fphar.2022.1060359

**Published:** 2022-11-23

**Authors:** Laia Caja, Enza Lonardo, Patricia Sancho

**Affiliations:** ^1^ Department of Medical Biochemistry and Microbiology, Science for Life Laboratory, Biomedical Center, Uppsala University, Uppsala, Sweden; ^2^ Institute of Genetics and Biophysics (IGB-CNR), Naples, Italy; ^3^ Translational Research Unit, Hospital Universitario Miguel Servet, IIS Aragon, Zaragoza, Spain

**Keywords:** cancer stem cells, cancer metabolism, tumor microenvironment, pancreatic ductal adenocarcinoma, glioblastoma, hepatocellular carcinoma

Cancer accounted for nearly 10 million deaths worldwide (i.e., one in six deaths) in 2020 (WHO). Although advances in diagnosis and treatment have led to significantly increased patient survival over the last decade for some cancer types, several highly aggressive cancers remain as unmet clinical needs. These cancers share common features, including resistance to radiotherapy and chemotherapy, rapid relapse after treatment, and metastasis formation. The presence of a specific subpopulation of malignant cells with tumor- and metastasis-initiating properties, such as cancer stem cells (CSCs), has been credited as the cause of these features.

Cancer is a dynamic disease, and the genomic instability within tumor cells gives rise to the genetic/epigenetic diversity underpinning tumor heterogeneity. Many intriguing questions about the spatiotemporal dynamics of this heterogeneity, and how it forms, are centered on the tumor microenvironment (TME). In the TME, several microenvironmental (external) and cell-autonomous (internal) factors, coupled with bidirectional cross-talk among cells, cooperatively promote stemness, tumor progression, metastasis, therapy resistance, and tumor relapse.

The scientific community has tried for decades to understand cancer behavior in the context of therapeutic strategies. The idea that cancer aggressiveness may rely on CSCs that share features of normal stem cells has changed the perspective with regard to new therapeutic approaches. In this regard, the study of the TME (or CSC niche) has become essential, due to the TME’s capacity to induce metabolic reprogramming of CSCs (e.g., adaptation to hypoxia, nutrient deficiency, acidosis, and reactive oxygen species). These unique metabolic features of CSCs make them essentially different from normal stem cells and also represent excellent potential therapeutic targets.

This article collection highlights the essential role of cellular metabolism for prognosis, tumor progression, and CSC targeting in aggressive cancer types such as melanoma, pancreatic adenocarcinoma (PDAC), hepatocellular carcinoma (HCC), and glioblastoma (GBM). This issue includes four articles representing the work of more than 30 authors in the field of cancer research.

Amino acids are vital nutrients for the survival of all cell types; in cancer, their metabolism is reprogrammed to facilitate the proliferation of cancer cells under stress conditions. Moreover, amino acid derivatives contribute to the immune responses and epigenetic regulation observed in tumor progression. As Chisari et al. discuss in their review, both glucose and amino acid metabolism are rewired to meet cancer cells’ needs as they are challenged with lack of nutrients during tumor growth. The review focuses on PDAC, HCC, and GBM, three of the most aggressive types of tumor. Cancer cells from different origins can demonstrate specific amino acid requirements and become dependent on non-essential amino acids, which are therefore termed conditionally essential amino acids. One example is glutamine, which is essential for proliferation, redox regulation, and purine and pyrimidine biosynthesis. Similarly, the metabolism of tryptophan, an essential amino acid, supports not only tumor cells but also stromal cells, which use its byproducts for their own nucleotide synthesis; moreover, the expression of the enzymes that metabolize tryptophan has been correlated with worse prognosis and immune suppression. These and many other examples are reviewed in Chisari et al.; ultimately, they emphasize that targeting amino acid metabolism has the potential to improve the survival of patients suffering from the most aggressive cancer types.

Cecchi et al. demonstrate how treatment with dexamethasone, a synthetic glucocorticoid, could be detrimental in patients with melanoma. They report that dexamethasone induced the expression of tryptophan 2,3-dioxygenase (TDO), the enzyme responsible for the conversion of tryptophan to kynurenine, and TDO was then found responsible for the induction of stemness properties in melanoma cells. This article highlights the need to better understand the effects of dexamethasone in melanoma, as TDO is known to promote immune escape, and as these authors have now shown, it can also promote cancer stem cell properties.

Over the past few years, a growing number of articles have described the diverse mechanisms by which a hypoxic TME promotes stemness in different cancer types. This is especially relevant in PDAC, where strong desmoplasia impairs nutrient and oxygen distribution, creating a starved and hypoxic microenvironment. Tian et al. have identified a novel hypoxia/stemness-based gene signature with prognostic value in PDAC, composed of eight genes (*JMJD6*, *NDST1*, *ENO3*, *LDHA*, *TES*, *ANKZF1*, *CITED*, and *SIAH2*). Most interestingly, the authors pinpoint an association between risk scores and the expression of immune checkpoint genes, suggesting that patients in the low-risk group might be more likely to respond to immune checkpoint inhibitors. These results reinforce the connection between tumor metabolism, CSCs, and the immune system, as revealed by bioinformatic analyses, a discipline with increasing significance for cancer research.

The study of lipid metabolism in the context of cancer stemness and aggressiveness has attracted a lot of attention in recent years. The review by Hu et al. highlights recent advances in this field, including the role of lipid uptake and storage in lipid droplets, lipid catabolism through lipolysis and fatty acid oxidation, modifications through the desaturation or peroxidation of CSCs, as well as the influence of lipid signaling on the CSC niche. In general terms, with the exception of lipid peroxidation, lipid metabolism supports stemness and progression and correlates with poor prognosis in a variety of cancers. However, the role of lipid metabolism in normal stem cells, as well as the heterogeneity of CSCs in different cancer types, remains poorly understood. The authors also identify targeted delivery, the side effects of lipid-targeting drugs, extracellular signaling based on lipids as future avenues of research.

In conclusion, the articles included in this topic reflect the evolution of the field of cancer metabolism in recent years, highlighting the tumor cells’ use of alternative substrates such as aminoacids and lipids over glucose and the key role of metabolism in regulation of the immune system.

